# Leveraging a health information exchange for analyses of COVID-19 outcomes including an example application using smoking history and mortality

**DOI:** 10.1371/journal.pone.0247235

**Published:** 2021-06-03

**Authors:** Guillermo A. Tortolero, Michael R. Brown, Shreela V. Sharma, Marcia C. de Oliveira Otto, Jose-Miguel Yamal, David Aguilar, Matt D. Gunther, Dania I. Mofleh, Rachel D. Harris, Jemima C. John, Paul S. de Vries, Ryan Ramphul, Dritana Marko Serbo, Jennifer Kiger, Deborah Banerjee, Nick Bonvino, Angela Merchant, Warren Clifford, Jenny Mikhail, Hua Xu, Robert E. Murphy, Qiang Wei, Farhaan S. Vahidy, Alanna C. Morrison, Eric Boerwinkle

**Affiliations:** 1 School of Public Health, University of Texas Health Science Center at Houston, Houston, TX, United States of America; 2 Harris County Public Health, Houston Texas, United States of America; 3 City of Houston Health Department, Houston, Texas, United States of America; 4 Greater Houston Healthconnect, Houston, Texas, United States of America; 5 School of Biomedical Informatics, University of Texas Health Science Center at Houston, Houston, TX, United States of America; 6 Center for Outcomes Research, Houston Methodist, Houston, Texas, United States of America; University of Oxford, UNITED KINGDOM

## Abstract

Understanding sociodemographic, behavioral, clinical, and laboratory risk factors in patients diagnosed with COVID-19 is critically important, and requires building large and diverse COVID-19 cohorts with both retrospective information and prospective follow-up. A large Health Information Exchange (HIE) in Southeast Texas, which assembles and shares electronic health information among providers to facilitate patient care, was leveraged to identify COVID-19 patients, create a cohort, and identify risk factors for both favorable and unfavorable outcomes. The initial sample consists of 8,874 COVID-19 patients ascertained from the pandemic’s onset to June 12^th^, 2020 and was created for the analyses shown here. We gathered demographic, lifestyle, laboratory, and clinical data from patient’s encounters across the healthcare system. Tobacco use history was examined as a potential risk factor for COVID-19 fatality along with age, gender, race/ethnicity, body mass index (BMI), and number of comorbidities. Of the 8,874 patients included in the cohort, 475 died from COVID-19. Of the 5,356 patients who had information on history of tobacco use, over 26% were current or former tobacco users. Multivariable logistic regression showed that the odds of COVID-19 fatality increased among those who were older (odds ratio = 1.07, 95% CI 1.06, 1.08), male (1.91, 95% CI 1.58, 2.31), and had a history of tobacco use (2.45, 95% CI 1.93, 3.11). History of tobacco use remained significantly associated (1.65, 95% CI 1.27, 2.13) with COVID-19 fatality after adjusting for age, gender, and race/ethnicity. This effort demonstrates the impact of having an HIE to rapidly identify a cohort, aggregate sociodemographic, behavioral, clinical and laboratory data across disparate healthcare providers electronic health record (HER) systems, and follow the cohort over time. These HIE capabilities enable clinical specialists and epidemiologists to conduct outcomes analyses during the current COVID-19 pandemic and beyond. Tobacco use appears to be an important risk factor for COVID-19 related death.

## Introduction

Since March 2020, much has been learned about the epidemiology of the novel coronavirus (SARS-CoV-2), which causes the Coronavirus Disease 2019 (COVID-19). However, much remains unclear, especially regarding why some may be asymptomatic while others may develop severe or fatal complications. Widely agreed upon factors that predict poor outcomes include advanced age and male gender, while a whole host of other risk factors have been suggested but thus far have insufficient evidence for their association [1–6]. It is likely that such predictors vary by gender and race/ethnicity. Identifying and characterizing predictors of adverse and positive outcomes requires large sample sizes with rich demographic, lifestyle and clinical data, ethnic and social diversity, and longitudinal follow-up.

The greater Houston area is one of the largest and most diverse regions of the United States with a population of over seven million individuals and an area that covers over 9,000 square miles [7]. Houston has been touted in both popular and scientific literature as how the demographic makeup of the United States may look in the future [7]. The greater Houston area has a large well-integrated health information exchange (HIE) that coordinates patient care by facilitating sharing of healthcare data among healthcare providers to coordinate patient care. We describe here the creation of a large COVID-19 patient cohort using data from the HIE to facilitate outcomes analyses, quality of care improvements, and assistance to public health agency operations. As an example application, we provide an analysis examining the association between tobacco use and COVID-19 fatality.

## Methods

### Cohort creation and data collection

Greater Houston Healthconnect (GHH) is a Texas based regional HIE founded in 2010 with the mission of enabling care coordination across an entire community through a secure network of electronic health record systems (EHRs), linking over 1,500 venues of care in greater Houston, South and East Texas, and Western Louisiana. This connectivity at the point of care improves quality, efficiency, and patient safety. Building on this platform further enables additional services for population health management, public health, and health services research. GHH is connected to over 95% of hospitals and health care systems in the greater Houston area and the majority of practicing physicians, representing over 15 million unique patients, making it one of the largest HIEs in the US.

**Fig 1:**
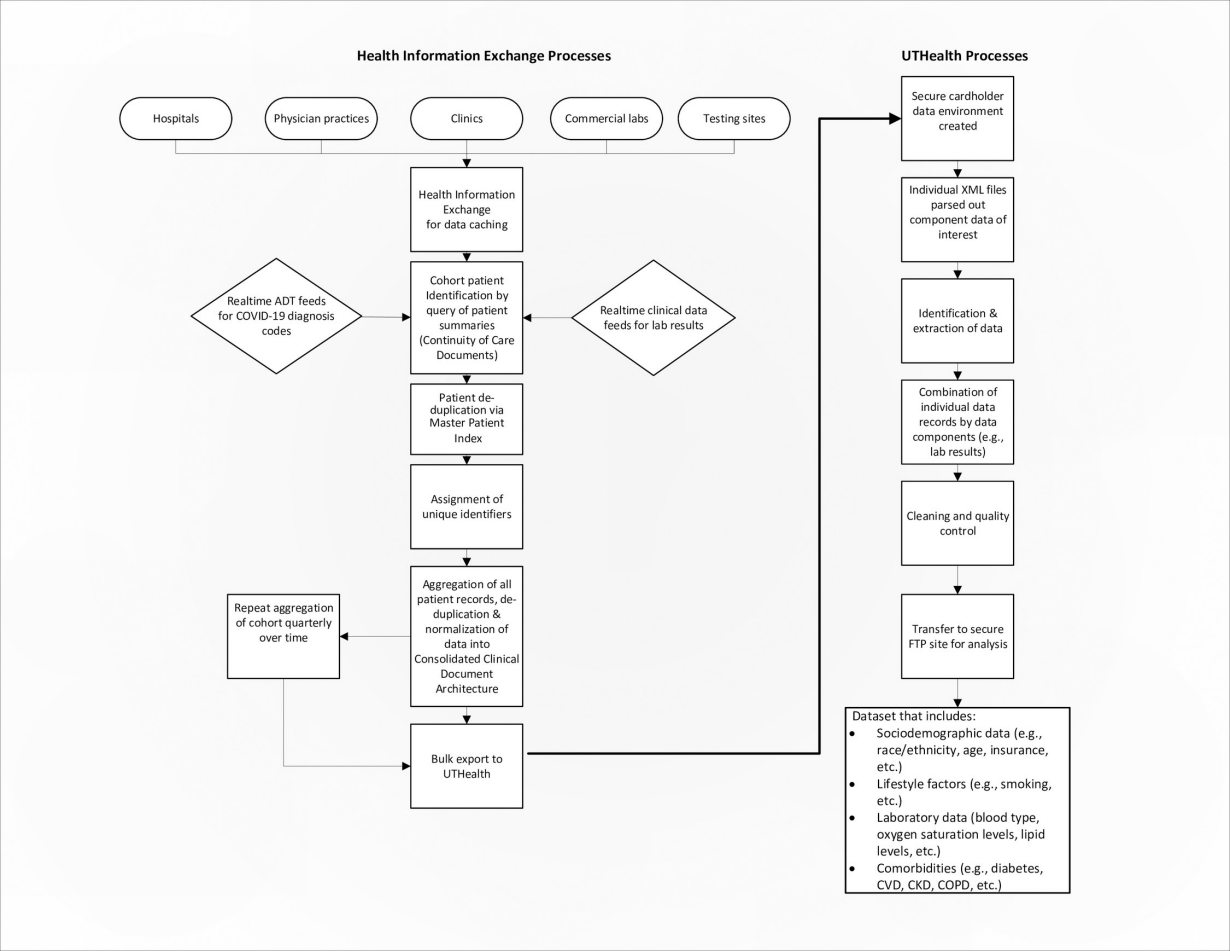
Data extraction and processing.

GHH has multiple interfaces with each participating provider’s EHR system. The first is an Admission, Discharge, Transfer (ADT) interface that feeds messages in real-time to populate its master patient index (MPI). The second is a two-way interface which enables the exchange of clinical information unique to the participant’s EHR. The third set of interfaces feeds discrete clinical data from laboratory, pathology, radiology, and other ancillary systems in real-time. Transcribed reports including progress notes, history and physicals, discharge summaries, and other text-based documents are also included. These interfaces in addition to GHH’s diagnostic imaging exchange service allow for the real-time monitoring of patient encounters and the collection of their clinical and demographic data, documents, and diagnostic images. Fig 1 displays the extract, transform and load (ETL) processes associated with data curation, validation and preparation.

Patients with specific diagnostic codes can be identified and queried for their patient records. To create this COVID-19 cohort, GHH surveilled acute, ambulatory, and post-acute providers for international classification of disease version 10 (ICD-10) diagnosis codes B97.21—SARS-associated coronavirus as the cause of diseases classified elsewhere; B97.29—other coronavirus as the cause of diseases classified elsewhere; and U07.1—COVID-19, which became available on April 1, 2020. Patients were entered into the registry for the first freeze of the cohort if they had these COVID-19 diagnosis codes or positive lab results from onset to June 12^th^, 2020. Patients in this registry were queried and their medical records were returned for aggregation in a clinical data repository (see Fig 1).

Identification and creation of the COVID-19 cohort is only the initial step in creating the infrastructure in support of outcomes and health services research. The second step is the assimilation and harmonization of phenotypic data collected across disparate healthcare systems to obtain the necessary demographic, lifestyle, laboratory, and clinical data, including outcomes or endpoint data. Clinical outcomes were ascertained according to International Classification of Disease version 10 (ICD-10) and Current Procedural Terminology (CPT) codes, allowing for standard harmonization across the HIE. The categories of data collected are displayed in Fig 1. For the purpose of this study, we examined the association between tobacco use and COVID-19 fatality.

The primary outcome was whether the patient was deceased or alive on June 12^th^ as identified in the data. The primary risk factor studied was the patient’s tobacco use history categorized as current or former tobacco users compared to never tobacco users. The patients’ tobacco history was determined from social history entries that included smoking status and history of tobacco use. Covariates examined included age, gender, body mass index (BMI), number of comorbidities, race/ethnicity categorized as White, Black, Hispanic/Latino, and Other. Comorbidities included history of hypertension, asthma, diabetes, chronic obstructive pulmonary disease (COPD), cardiovascular disease (CVD), pre-CVD, chronic kidney disease (CKD), ischemic stroke, and congestive heart failure, and a count of comorbidities for each individual was used for the analyses presented here [2, 3, 8–12]. The outcomes and quality improvement analyses were reviewed and approved by multiple institutional review boards and committees for the protection of human subjects, including the University of Texas Health Science Center at Houston and the Western Institutional Review Boards. Statistical analyses were done using anonymized data. The participating institutional review board had waived the need for informed consent for these outcomes and quality improvement analyses.

### Statistical analysis

Descriptive data were examined for outliers and missing information. The initial sample consisted of 8,874 patients with a COVID-19 diagnosis. Patients that were less than 18 years of age were excluded from the smoking—mortality analysis (N = 319). The multivariable logistic regression analysis was restricted to 5,285 individuals with complete data for the independent and dependent variables of interest as well as covariates. Characteristics of COVID-19 patients that survived and those who died were compared using χ^2^ test and Student’s t-test. Statistical significance was defined by using a nominal p-value of 0.05 adjusted for the number of tests conducted. Multivariable logistic regression models were constructed to examine how each covariate affected the association between tobacco use and fatality, controlling for other covariates. The final model included variables that were significantly associated with COVID-19 fatality or that affected the association between smoking status and COVID-19 fatality. No attempt was made to account for clustering by county or healthcare system because patients move among counties and healthcare systems, which is an advantage of using an HIE for cohort collection.

## Results

The research team was able to rapidly access, extract, and clean data ready for analysis from the HIE. The extracted data contained demographic, clinical, and laboratory data on a total of 8,874 patients with diagnoses codes or positive test lab results for SARS-CoV-2. Table 1 shows patient characteristics for the entire sample and by non-fatality and fatality. The sample consisted of 45.3% males, 25.4% Black patients, and 22% Hispanic/Latino patients (see Table 1). The mean age in the sample was 50.3 years (SD = 19.64) and ranged from 0 years to 101 years of age.

**Table 1:** Descriptive findings of patients in cohort.

Variable	No death	Deaths	Total	Unadjusted Odds Ratio (95% CI)	P-Value
	(n = 8,399)	(n = 475)	(n = 8874)		
**Age** (years) $\bar{x}$ (SD)	49.15 (19.27)	70.87 (13.78)	50.32 (19.64)	1.07 (1.06, 1.08)	<0.001
**Gender**					<0.001
Female	4,667 (55.58%)	188 (39.58%)	4,855 (54.72%)	1.00 (ref)	
Male	3,730 (44.42%)	287 (60.42%)	4,017 (45.28%)	1.91 (1.58, 2.31)	
**Race/Ethnicity**					0.003
White	2,810 (33.46%)	86 (39.16%)	2,996 (33.76%)	1.00 (ref)	
Black	2,125 (25.30%)	132 (27.79%)	2,257 (25.43%)	0.94 (0.75, 1.18)	
Hispanic/Latino	1,870 (22.26%)	83 (17.47%)	1,953 (22.01%)	0.67 (0.51, 0.87)	
Asian	286 (3.41%)	19 (4.00%)	305 (3.44%)	1.00 (0.75, 1.18)	
Other	1,308 (15.57%)	55 (11.58%)	1,363 (15.36%)	0.64 (0.47, 0.86)	
**BMI** (kg/m^2^)	30.97 (8.04)	30.92 (9.63)	30.97 (8.14)	0.99 (0.98, 1.01)	0.910
**Number of Comorbidities** $\bar{x}$ (SD)	0.830 (1.34)	1.66 (1.80)	0.87 (1.38)	1.39 (1.32, 1.47)	<0.001
**Tobacco History**					<0.001
Never	3,808 (75.18%)	161 (55.33%)	3,969 (74.10%)	1.00 (ref)	
Current or Former	1,257 (24.82%)	130 (44.67%)	1,387 (25.90%)	2.45 (1.93, 3.11)	

Data are mean (SD), n (%). p values were calculated by Student’s t-test, χ^2^ test. Age (n = 8,874), Gender (n = 8,872), Race/Ethnicity (n = 8,874), BMI (n = 5,103), Number of Comorbidities (n = 8,836), Tobacco Use History (n = 5,285). Significance at p<0.05.

Information on tobacco use history was available for 5,285 patients 18 years or older in the cohort. There were significant differences between patients with and without tobacco use history by age, gender, and race/ethnicity. Those with missing data on tobacco use were more likely to be younger ($\bar{x}$ = 47.76 years vs $\bar{x}$ = 50.32 years), male (49.03% vs 45.28%), and a member of the ‘Other’ race category (26.18% vs 15.36%).

One in four (25.90%) patients had a former or current history of tobacco use, which is higher than the state average [13]. Those who died from COVID-19 had significantly greater odds of tobacco use as compared to those who did not die (OR = 2.45; 95% CI 1.93, 3.11; p-value < 0.001). Male gender, race/ethnicity, increasing age, and increasing number of comorbidities were all significantly associated with fatality.

Table 2 displays the results of multivariable logistic regression for the association between tobacco history and fatality. After controlling for age, the association between tobacco use and fatality remained significant (AOR = 1.73; 95% CI 1.34, 2.22; p-value < 0.001). After controlling for age, gender, race/ethnicity, and number of comorbidities the odds of fatality among patients with a history of tobacco use was 1.54 (95% CI 1.19, 1.99; p-value < 0.001) times greater as compared to fatality among those without a history of tobacco use.

**Table 2:** Multivariable Logistic Analysis between risk factors associated with COVID-19 fatality.

Variable	Model 1	Model 2	Model 3	Model 4
	(n = 5,285)	(n = 5,285)	(n = 5,285)	(n = 5,285)
**Tobacco Use History**				
** **Never	1.00 (ref)	1.00 (ref)	1.00 (ref)	1.00 (ref)
** **Current or Former	1.73 (1.34, 2.22)	1.59 (1.23, 2.06)	1.65 (1.27, 2.13)	1.54 (1.19, 1.99)
**Age** (per year)	1.07 (1.06, 1.08)	1.07 (1.06, 1.08)	1.08 (1.07, 1.08)	1.07 (1.06, 1.08)
**Gender**				
** **Female		1.00 (ref)	1.00 (ref)	1.00 (ref)
** **Male		1.58 (1.22, 2.03)	1.60 (1.24, 2.06)	1.60 (1.24, 2.07)
**Race/Ethnicity**				
** **White			1.00 (ref)	1.00 (ref)
** **Black			1.39 (1.02, 1.89)	1.25 (0.91, 1.70)
** **Hispanic/Latino			1.72 (1.22, 2.43)	1.65 (1.16, 2.33)
** **Asian			1.06 (0.51, 2.21)	1.09 (0.52, 2.27)
** **Other			1.35 (0.81, 2.23)	1.36 (0.82, 2.26)
**Number of Comorbidities**				1.21 (1.12, 1.30)

Data are odds ratios (95% CI) p values were calculated by multivariable logistic regression. Age (n = 8,874), Gender (n = 8,872), Race/Ethnicity (n = 8,874), BMI (n = 5,103), Number of Comorbidities (n = 8,836), Tobacco Use History (n = 5,285). The likelihood ratio test x^2^ = 417.36; p-value < 0.001.

## Discussion

We leveraged a regional HIE to create a cohort of COVID-19 patients for the purpose of outcomes and health services research aimed at quality improvement. The effort was a partnership among the HIE and its provider participants, academia, and regional public health authorities. This collaboration enabled the creation of one of the largest cohorts of COVID-19 patients and their medical histories across the continuum of care. The first cohort, used for this study, consisted of 8,874 COVID-19 patients identified from the pandemic’s onset in the region through the first peak and decline. A second cohort, continued after the cohort one freeze, has captured the next rise and peak cycle and will consist of over 200,000 COVID-19 patients. HIEs offer a relatively quick and efficient means to acquire large data sets to identify potential risk factors of emerging diseases. Additionally, HIEs offer researchers access to a wide array of lifestyle, clinical, laboratory, and demographic characteristics to conduct exploratory and confirmatory studies so public health officials can intervene and improve population health. There were many components to consider when leveraging an HIE to create a cohort of COVID-19 patients with their complete medical history for outcomes investigations. A strong partnership among the private sector HIE, healthcare providers, academia, and local governmental public health agencies must be developed. Each of these groups brings unique perspectives, tools, and strengths to the overall effort. The HIE was able to rapidly identify retrospective demographic information and comorbidities. Public health authorities enabled the reporting and collection of cases and associated patient health information. Healthcare providers assisted in the interpretation of clinical and laboratory data. While, academia brought a population health perspective, as well as relevant statistical methods, data analytics, and geospatial visualization.

HIEs have been successfully leveraged for outcomes research such as diabetes, cardiovascular disease, and other outcomes [1]. In this study, we demonstrate how a HIE was accessed and utilized to study the association of tobacco use history and COVID-19 fatality. Previous studies have shown mixed evidence regarding the association between tobacco use and poor outcomes in COVID-19 patients [14, 15]. A rapid evidence review using Bayesian meta-analysis techniques identified 50 studies examining the association between smoking status and COVID-19 mortality, but only nine studies were of sufficient quality to include in the analysis [14]. Most previous studies had small samples and lacked ethnic/racial diversity. This meta-analysis reported former smokers had an increased risk of mortality compared to never smokers, but reported inconclusive results for the risk in current smokers. We report here that tobacco use is significantly associated with COVID-19 fatality (OR = 2.45), and the association remained significant after controlling for age, gender, race/ethnicity, and number of comorbidities.

The potential relationship between tobacco use and COVID-19 fatality may be explained by many factors. It is well established that smoking is a risk factor for respiratory infections and other viruses such as influenza [16]. Evidence suggests that tobacco use reduces the immune response, impairing patients’ ability to fight infection. Tobacco use is also associated with other known COVID-19 risk factors such as chronic obstructive pulmonary disease, diabetes, and heart disease [17]. It has also been found that smoking tobacco may cause an increase in the levels of angiotensin-converting-enzyme-2 (ACE2) protein, which has been shown to be the entry receptor for SARS-CoV-2 [17, 18]. Tobacco use clearly weakens lung function, and can lead to increased epithelial permeability, as well as oxidative stress [17]. The results of this study add to the growing evidence that tobacco use is associated with COVID-19 related fatality.

This study has several limitations. The cohort consisted of patients who had EHR data available from participating hospitals and providers in the HIE and may not represent the general population. However, it is important to note that greater than 95% of the hospital systems in the greater Houston area participate in the HIE. Since the patient population came mainly from providers and hospital systems, patients in the cohort may be sicker than a random sample of SARS-CoV-2 individuals in the general population. Also, patient records were extracted early in the pandemic before widespread testing was implemented. Therefore, patients captured in this first freeze may be more symptomatic than patients seen at later dates. Since data came from EHRs, a large number of patients did not have data on history of tobacco use and specific data on frequency of tobacco use were not available. Our study also does not elucidate various mechanistic pathways that may explain association between tobacco consumption and poor COVID-19 outcomes. Finally, in the use case provided we did not adjust for detailed disease severity parameters, lab values, medications and other treatment characteristics of COVID-19 patients. Our ongoing efforts in data harmonization will permit such analyses in future iterations of HIE data.

Public health uses of HIE, such as mandated reporting of laboratory diagnoses and physician-based diagnoses have been widely studied [19]. During the COVID-19 pandemic, the importance of healthcare informatics infrastructure has been well recognized [20]. Issues such as privacy for health information exchange and research uses have been discussed as well [21]. Nevertheless, this is one of the first studies that leverages an existing large HIE to support evidence generation for COVID-19. In the future, we will further improve the quality and standardization of HIE data to support more efficient COVID-19 studies [19].

## Conclusion

HIEs can be a vital resource to more rapidly ascertain a large population of COVID-19 patients and their demographic information, lifestyle factors, and medical history, and to conduct outcomes research. This study demonstrates how a potential risk factor for COVID-19 fatality can be quickly examined to inform public health and medical professionals. Our findings indicate that a history of tobacco use is associated with death in patients with COVID-19 after controlling for age, gender, race/ethnicity and the number of comorbidities. Tobacco use is a known risk factor for many illnesses including respiratory illnesses, cardiovascular diseases, cancer, asthma, diabetes, and COPD [15, 16]. Given that existing research on the association between tobacco use and COVID-19 outcomes are varied, this research builds on the evidence that tobacco use may be an important risk factor for COVID-19 fatality.
